# Differences and Similarities in the Feet of Metatarsalgia Patients with and without Rheumatoid Arthritis in Remission

**DOI:** 10.3390/jcm13102881

**Published:** 2024-05-14

**Authors:** Rebeca Bueno Fermoso, Maria Rosario Morales Lozano, Marta Nieto Cordero, Carmen Martínez Rincón, Pablo García-Fernández, María Luz González Fernández

**Affiliations:** Faculty of Nursing, Physiotherapy and Podiatry, Complutense University of Madrid, 28040 Madrid, Spain; rebueno@ucm.es (R.B.F.); martni05@ucm.es (M.N.C.); nutrias@enf.ucm.es (C.M.R.); pablga25@ucm.es (P.G.-F.); luzalez@ucm.es (M.L.G.F.)

**Keywords:** foot funtion index, foot, forefoot, metatarsalgia, metatarsal, osteoarticular damage, rheumatoid arthritis, rheumatoid arthritis in remission, synovitis, ultrasound

## Abstract

**Objectives:** Metatarsalgia continues to be a problem in patients with rheumatoid arthritis (RA) in remission (remRA), as well as in the non-rheumatic population, with a mechanical origin. Identify and compare clinical, morphological, disability, synovitis (ultrasound), and radiological osteoarticular damage characteristics in two groups of patients with lesser-ray metar-tarsalgia, with remRA, and without RA. **Methods:** Cross-sectional study with 84 patients with remRA (mRA) and 60 patients without RA (nmRA). The study evaluated five clinical variables (pain, Foot Function Index (FFI), joint mobility, digital deformities, and foot type), a radiological variable (osteoarticular damage), and an ultrasound variable (metatarsal synovitis). The data were analysed using descriptive and correlational techniques. **Results:** There were no significant differences in sex, age, body mass index (BMI), or degree of pain. Both groups showed a high prevalence of limited joint mobility for the ankle and first metatarsal phalanx (DF1st MTPJ) and digital deformities, with no statistically significant differences. Similarly, there were no differences in lesser-ray synovitis. On the other hand, there were differences in mRA with greater disability and activity limitation (FFI), LDD (lesser-ray digital deformities) stiffness, first-ray deformities, radiological damage, synovitis in 1st MTPJ, and positive Doppler (five patients). **Conclusions:** Metatarsalgia presents similarities in both populations. Biomechanical factors may influence the symptoms and presence of synovitis in patients with RA in remission. Other characteristics are more frequent in mRA, which could be related to the disease; thus, future research should include both biomechanical and ultrasound exploration of the foot in the valuation of patients in remission.

## 1. Introduction

Rheumatoid arthritis (RA) is an autoimmune, inflammatory, chronic, and progressive disease. It has a prevalence of 0.3–1.5% in the world population [1]. According to European prevalence data, it occurs in 0.20% to 0.40% of the European population [2]. While in Spain, data estimates range from 0.3 to 1.6% [3]. It is more frequent in women aged 30–50 years old [4]. The essential sign of RA is the destructive capacity of synovial inflammation [5]. 

The feet are among the most affected anatomical regions, generating osteoarticular symptoms, deformities, and affectation in up to 80–90% of cases [6,7,8,9,10]. Its effects may involve disability [11,12,13,14,15,16] and limitations on the activities of daily living [17], with negative effects on quality of life [18]. 

Within the foot, the forefoot is one of the most frequently involved areas, affecting 70–94.3% of patients, and it is, along with the ankle, one of the first areas to become affected [9,17,19]. The main deformities found in the feet are subluxation of the lesser metatarsophalangeal joints (MTPJ) [20], hallux valgus (HV) [6,7,8,15,19,21,22,23,24,25], hallux limitus (HL), hallux rigidus (HR), and greater prevalence of flat feet or pronation [7,19,21,23,26,27,28]. Osteoarticular damage includes erosion and joint space reduction (JSR), with MTPJ being the most affected [11,29]. This variable is used in the osteoarticular damage evaluation scales along with the metacarpophalangeal joints. 

Despite advances in current treatments, through the use of biological therapies [30], patients in remission, in many cases, continue to present pain, frequently in the foot [31,32,33], and specifically in the forefoot [32,33,34]. Moreover, osteoarticular deformities and affectation increase with the evolution of the disease [26,33,35,36]; thus, it is important to determine and control the causal mechanisms involved in order to use the therapeutic window. 

Metatarsal pain is also common in the non-rheumatic population, with a prevalence of 10% [37] in the population and 32.5–88.6% [38,39] in specialised medical practices, identifying its aetiology as structural and biomechanical in 84–99.6% of cases [37,38,39,40]. Furthermore, this metatarsal pain constitutes a risk factor for motor disability [41] and a decreased quality of life [42], although improvements in symptomatology are possible for these patients through both orthopodologic [43] and surgical [37] treatments. 

The metatarsal pain of patients with RA in remission may be influenced by structural and biomechanical factors; in fact, cases of plantar orthosis have shown improvement in metatarsal pain symptoms in patients with RA [44]. Determining the similarities and differences between metatarsalgia in patients with and without RA may help identify the underlying factors that may influence metatarsal pain. This could lead to the development of a more adequate treatment.

If this is the case, the study will provide knowledge that can help in the identification of biomechanical factors that may interfere with pain in patients with RA in remission, opting in the first instance for conservative orthopaedic treatments to reduce their symptoms and improve their quality of life, as is achieved in patients without RA. 

The objectives of this paper are as follows: to identify and compare clinical and radiological differences, as well as the presence of synovitis, between patients with lesser-ray metatarsalgia, with RA in remission, and without RA.

## 2. Materials and Methods

### 2.1. Study Design and Participants

#### 2.1.1. Study Design

A cross-sectional study of cases and controls was carried out. The study was conducted according to the protocol and principles of Good Clinical Practice and the 2005 Declaration of Helsinki, and it was approved by the Ethics Committee of Medical Research of San Carlos University Clinical Hospital of UCM on 21 December 2021, in Madrid (Spain), 21/719-E.

#### 2.1.2. Context

The evaluation of the patients was carried out in the Rheumatic Foot Unit of the University Clinic of Podiatry at the Universidad Complutense de Madrid (UCM) from January 2022 to May 2023. 

The patients were evaluated in a single day in three different medical offices within the clinic. The study lasted a maximum of 1.5 h and was performed by three different professionals. A fourth professional evaluated the X-rays afterwards. 

In the first room, a first researcher explained the study to the patient, identified them, conducted part of the clinical interview to classify the group to which they belonged, and applied the primary inclusion or exclusion criteria, making them sign the informed consent form. 

Patients who met the first inclusion criteria and signed the informed consent form were sent to a second room, where a second researcher proceeded to collect data. Firstly, by means of a clinical interview, the degree and exact location of the pain were asked, and both were evaluated, but only the foot with pain or with more pain in the case of bilateral pain in the lesser metatarsals was used for the study. They carried out the rest of the clinical interview and clinical examination and collected the radiographs to serialise them and provide them to a fourth researcher. 

Once this had finished, they accessed a third room where a third researcher was located, who blindly evaluated the patient’s metatarsophalangeal joints ultrasonographically, indicating the presence or absence of synovitis. 

A fourth researcher, who did not know the type of patient, received the anonymised radiographs and evaluated them according to the register of variables designed for the study. 

#### 2.1.3. Participants

The study population consisted of older men and women with less metatarsal pain.

The participants of the mRA group were referred by the rheumatology services of different public hospitals in Madrid (Spain), and the participants of the control group were recruited from the staff of the hospitals and university. 

The patients with RA had a diagnosis of RA based on the classification criteria established by the American College of Rheumatology and the European Alliance of Associations for Rheumatology in 2010 [45] and were in remission according to the DAS 28 [46] criteria (mRA group). The control group consisted of individuals with pain in their lesser MTPJs and no rheumatic diseases (nmRA group).

#### 2.1.4. Study Size

Sampling description: A minimum sample of 120 patients was needed to compare the prevalence of metatarsalgia in patients with rheumatoid arthritis and in patients without rheumatoid arthritis with a 95% confidence level and a minimum clinically important difference in proportions of 5%. 

Sample size: A total of 148 patients with metatarsalgia were recruited, adapted to the availability of patients referred by rheumatologists and the heterogeneity of the population by choosing 88 patients with RA and metatarsalgia and 60 controls with metatarsalgia and no RA. 

#### 2.1.5. Inclusion and Exclusion Criteria

The inclusion criteria were: metatarsalgia localised in the lesser metatarsals (2nd to 5th), and the provision of loading dorsoplantar and lateral radiographies taken in the last year. 

The exclusion criteria were as follows: patients who only had pain in the first MTPJ, patients under 18 years of age, patients with other rheumatic diseases, neuromuscular diseases, diabetes mellitus, ulcers, or skin tumour lesions, papilloma, history of trauma in the previous 6 weeks, osteoarticular surgery in the foot and/or ankle, undergoing orthopodological treatment, having been injected in the feet in the last three months, and individuals who could not collaborate in the surveys. 

After applying the inclusion and exclusion criteria, the patients were briefed on the procedure of the study to sign the informed consent form, and then the data were gathered. 

To ensure that the sample had no statistically significant differences in terms of age and sex, we first collected the sample of patients with RA, estimating the type of patients required in terms of age and sex and reviewing the data every 2 months during the data collection. Subsequently, information was provided on the characteristics required for the control group sample, considering only patients with similar characteristics in terms of age and sex but without matching the sample. 

### 2.2. Data Collection and Procedure

#### 2.2.1. Sample Selection

A first researcher identified whether the patient had RA and whether the patient was in remission according to the patient’s rheumatologist’s report. It was also identified if the patient had pain in the metatarsal area in the case group. In the control group, the research identified whether they had metatarsal pain. Both groups met the inclusion and exclusion criteria, and the informed consent form was signed. 

A second researcher evaluated the forefoot area and identified if the pain was in the lesser metatarsal area (lesser MTPJ), thereby configuring the patients who would participate in the study. 

**Figure 1:**
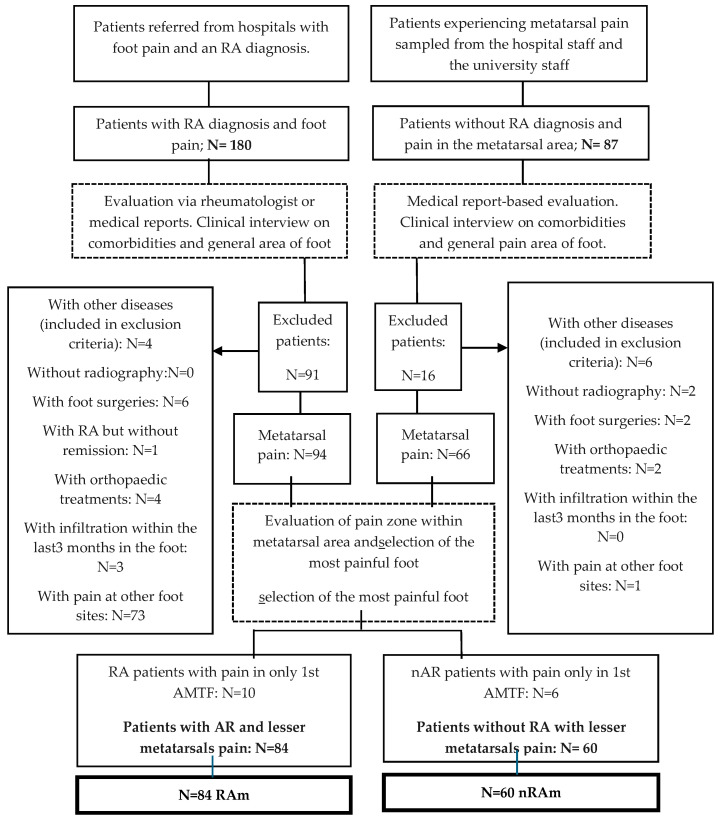
Flow chart.

A flow chart is provided in Figure 1, showing the process up to the selection of the final sample.

Once the patients who will form part of the sample have been selected, they will be examined and data will be collected. The study will consist of a clinical interview, a clinical examination, an ultrasound evaluation and the evaluation of the patient’s radiographs.

#### 2.2.2. Clinical Interview

The same researcher will conduct a clinical interview where he will collect: 

Sociodemographic data including weight, height, and foot size. 

Details related to the disease were gathered in the report of the patients (years of evolution of the disease, medication, and comorbidities). 

Habits related to smoking, alcohol and substance consumption, and activity level meet the criteria of a sedentary lifestyle based on the minimum recommendations of the WHO for each age and for chronic effects [47]. 

The VAS pain scale which is a scale where one indicates the lowest pain and ten indicates the greatest pain imaginable [48], to evaluate pain in each foot, gathering the data of the foot that presented the greatest pain. 

Pain was classified according to duration, chronic or acute (lasting more or less than 6 weeks, respectively) [49], and the type of pain, namely mechanical (during activity), inflammatory (pain at rest), mixed (a combination of both mechanical and inflammatory), or neurological (paraesthesia, hyperesthesia, and irradiation) [49,50].

The Foot Function Index questionnaire (FFI) [51,52], which consists of 23 items regarding the impact of foot impairments, has three subscales: foot pain (FFI pain) (nine items), foot disability (FFI disability) (nine items), and activity limitation (FFI limitation) (five items). This scale is widely used in patients with RA. Since these patients were not undergoing orthopodological treatment at the time of the study, the FFI pain study did not gather data related to pain with footwear or plantar orthosis. To obtain a subscale score, the values of each item were totaled and divided by the number of items considered applicable by the patient. By calculating the average of the three subscale scores, a total FFI score (FFI total) was produced. The values ranged between 0 and 100, where the higher values corresponded to greater pain, disability, and limitation. The questionnaire translated into Spanish FFI-sp [53] has shown excellent internal consistency, with the total of the items giving a Cronbach’s α 95%. By subscales, the values were 0.95 in that of pain, 0.96 in that of disability, and 0.69 in that of activity limitation.

#### 2.2.3. Clinical Examination

The same researcher will subsequently perform a clinical examination, which will consist in the assessment of joint mobility, digital deformities, and the evaluation of foot type.

- Joint mobility.

**Figure 2:**
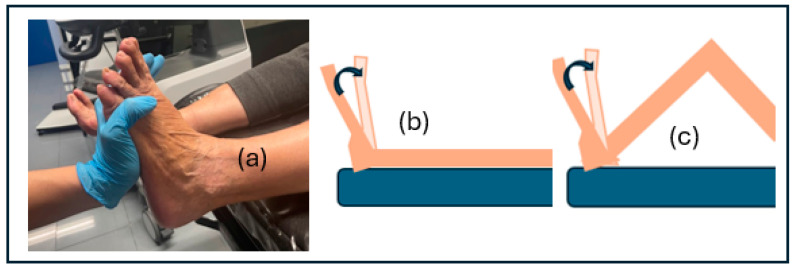
(**a**) supination of the foot. (**b**) Silfverskiöld’s test with an extended knee. (**c**) Silverskiöld test with a flexed knee.

The dorsiflexion of the ankle was evaluated using Silfverskiöld’s test (dorsiflexion of the tibiofibular-talar joint: DFTFTJ) [54], which explores the degree of dorsiflexion of the ankle in two positions: with an extended knee and with a flexed knee. The test is positive when dorsiflexion is limited with knee extension, which is corrected with flexion, and when there is limitation in both positions of the knee [54], considering both as limitations. The test is performed with supination of the foot; in this way, we could correct any possible pronation that would falsify the results [55]. According to the study of Pasarin Martínez A. et al. [55], the test has an intra-explorer reliability of 0.708–0.828. Figure 2 shows the image of the position of the foot and figures on the performance of the test with the knee in extension and flexion. 

Subtalar joint mobility was measured using a goniometer with the participant in the prone position. The zero starting position was defined as the position with the heel aligned with the midline of the tibia and the ankle joint in gentle dorsiflexion (that is, the Achilles tendon became taut) [56]. Normal movement is 30°. Limitation was considered when inversion mobility was below 20°.

**Figure 3:**
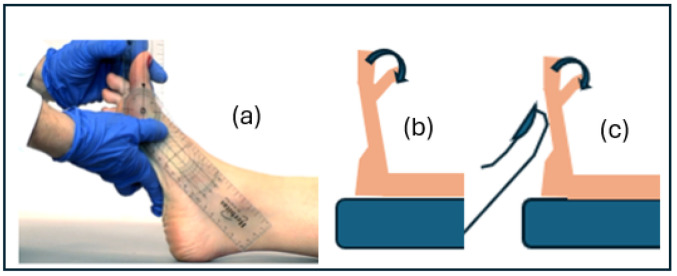
(**a**) Image of the mobility measurement using the Buell technique assisted by a goniometer. (**b**) Picture of the previous measurement (Buell technique). (**c**) Picture of measurement of the func-tional hallux limitus test according to the Dananberg technique.

DF1st MTPJ was evaluated through passive loading mobility [57] and the functional hallux limitus test (FHL test). Functional hallux limitus (HL) was considered when the patient presented a lack of movement under simulated loading in the first MTPJ (less than 20° degrees) but normal values in unloading, limited when it had less than 40° of dorsiflexion movement in unloading and a positive FHL test, and stiffness was considered when the patient showed a lack of movement at non-weight bearing. The normal range of motion of the first metatarsophalangeal joint is defined as being more than 65° dorsiflexion, while hallux rigidus is the severe limitation of hallux dorsiflexion (<30°). Sanchez Gomez et al. [57] found high reliability, quality, and CCI results with both measurements. Payne et al. [58] have reported that the FHL test has a sensitivity of 0.72 and a specificity of 0.66 as a predictor of abnormal foot pronation during the stance phase of the gait cycle. The limitations of the test are that it is not analysed dynamically and that the force performed by the examiner may not accurately represent that which the patient supported in dynamics. Figure 3 shows the image of the unloading test and two figures, showing the test performed in unloading and under simulated load.

- Digital deformities.

The presence or absence of digital deformities was recorded, differentiating between lesser toes and the first toe [59]. 

Lesser-toe digital deformities (lDD) were considered when the patient presented claw toe, hammer toe, mallet toe, or Tailor’s bunion. 

In the first toe, deformities were considered when the patient presented hallux valgus (HV), hallux limitus (HL), hallux rigidus (HR), or other (hallux varus, hallux extensus, hallux interphalangicus, and hallux flexus), and the degree of HV was measured through the Manchester scale, which considers four degrees of severity, although we only differentiated between grades I and II and between grades III and IV [60]. 

In the lesser-toe MTPJ, the presence or absence of subluxations and luxations was recorded [61], clinically counting their presence when there was a MTPJ affected. Subluxation or dislocation was evaluated in both the sagittal and transverse planes. 

The stiffness of lDD and MTPJ was valued through Kelikian’s test [62], which is widely accepted and consists of putting vertical pressure on the metatarsal heads at the plantar level and evaluating whether the plantar finger flexes and the deformity is corrected, differentiating between normality, semiflexibility, and stiffness. In flexible deformities, the MTPJ can be aligned; in semiflexible deformities, the MTPJ cannot be completely aligned; and in stiff deformities, the MTPJ does not change.

- Foot position.

Foot position was assessed using the Foot Posture Index (FPI). FPI [63], which assesses the position of the foot. In this validated method (ICC for the clinician, 0.94–0.96), each criterion is scored as −2, −1, 0, +1, or +2. The following cut-off points of FPI were used, which define the foot type category: (a) very supinated, −12 to −4; (b) supinated, −3 to 0; (c) neutral, 1 to 6; (d) pronated, 6 to 10; and (e) very pronated, 11 to 12, considering very pronated and pronated as pronated, and very supinated and supinated as supinated. 

#### 2.2.4. Ultrasound Scanning

For ultrasound scanning. A second researcher with a PhD in Podiatry, who specialised in rheumatic foot and had extensive experience in ultrasound, valued the anonymised patients. An ultrasound study was conducted, which included scoring synovitis based on a grey scale and Doppler power signal assessments in accordance with the Outcome Measures in Rheumatology Recommendations (OMERACT) [64], indicating only the presence or absence of synovitis for the dorsal aspect of the 1st, 2nd, 3rd, 4th, and 5th MTPJ. Ultrasound examinations were carried out by a single specialist using a Samsung 500 ultrasound system equipped with an 8 to 12 MHz linear probe. For the ultrasound examination, the patient was sitting with the knee flexed at 90° and the plantar side of the foot leaning on the stretcher. The researcher quantified the number of patients with some MTPJ with synovitis, the number of MTPJ with synovitis per foot both from 1st to 5th and from 2nd to 5th, and the total number of synovitis in the entire sample from 2nd to 5th MTPJ. 

#### 2.2.5. Radiological Assesment

**Figure 4:**
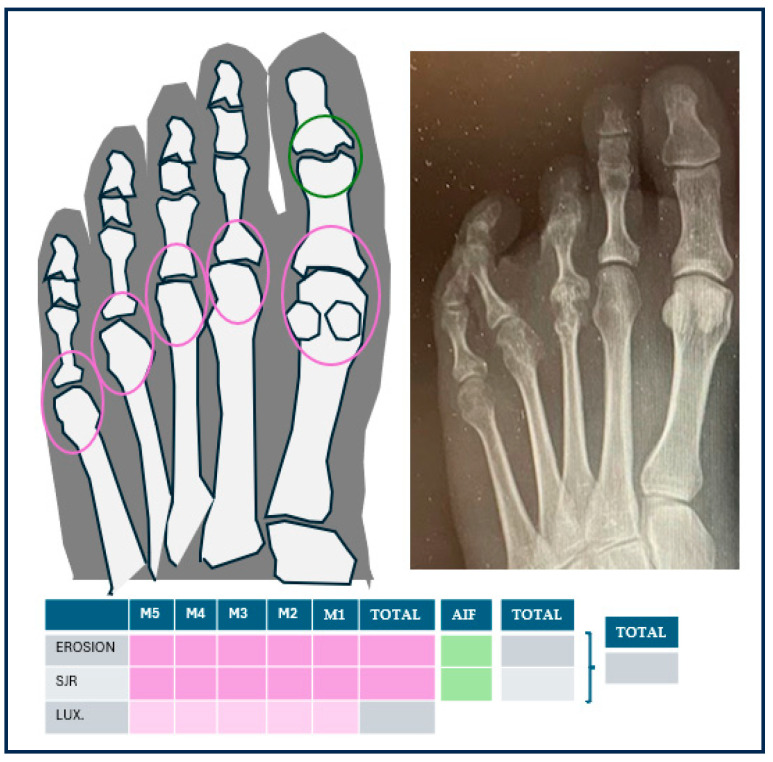
In the metatarsophalangeal joints (circled in pink) and the hallux interphalangeal joint (circled in green), the presence of erosions and decreased joint space were evaluated by summation. In addition, the presence of subluxation or dislocation of the metatarsophalangeal joints was indicated. On the right an example of a radiograph of a patient with rheumatoid arthritis.

The radiological assessment was carried out by a researcher with a PhD in Podiatry who specialised in rheumatic foot. This researcher evaluated the anonymised X-ray in loading dorsoplantar projection, provided by the patient. The 5th MTPJ and the first interphalangeal joint (IPJ) of the hallux were analysed, which are included in the scales of radiological damage [65]. To evaluate the osteoarticular damage, the researcher gathered: the presence or absence of erosions and JSR, assigning one point to each joint in the case of presenting erosions (regardless of their magnitude) and one point in the case of JSR (regardless of their degree), and the total score in each joint was evaluated (with a maximum of 2 points). The scores of the 6 joints were gathered [65], with a maximum of 12 points per foot. Moreover, the total amount of affected lesser-toe MTPJ was calculated, and the researcher specifically evaluated the number of affected MTPJ from 2nd to 5th. Luxation/subluxation of the MTPJ was also evaluated, which were defined as the partial (subluxation) or contactless (luxation) displacement of joint surfaces [66], considering also their presence or absence in each of the MTPJ and the total score. Figure 4 shows an image of an X-ray of a patient with metatarsal pain and a diagnosis of AR, a figure indicating the joints evaluated and a table in which the findings on the X-ray were filled in.

### 2.3. Bias

In order to avoid bias, the clinical examinations were always performed by the same researcher, a podiatrist with more than 10 years of clinical experience, as were the radiological and ultrasound examinations. The latter two were blinded, and the researchers were unaware of the presence or absence of RA and the location of the pain.

Dorsal ankle flexion, first metatarsophalangeal flexion, and loaded calcaneal position were assessed using a goniometer, and the qualitative value was then identified in order to avoid bias. 

### 2.4. Statistical Analysis

The data analysis was carried out using SPSS v25.0 (SPSS Inc., Chicago, IL, USA). The quantitative variables were defined as the mean and standard deviation. The discrete variables were defined as the number of cases and percentages. The normality of the distributions was explored using a Kolmogorov-Smirnov test. To compare and analyse the qualitative variables, the Chi-squared test was used. When the expected value was lower than 5 in any of the boxes of the contingency table, it was necessary to use Fisher’s exact test. To compare the independent quantitative variables, the Student’s *t*-test or Mann-Whitney U-test were used as a function of the data distribution. Statistical significance was established at *p* < 0.05.

## 3. Results

Initially, 267 patients were evaluated, including 180 patients with RA and foot pain and 86 patients without RA and metatarsal pain. Of these, 160 patients were eligible: 94 with RA and 66 without RA (causes of loss are provided in the flow chart, see Figure 1). 

Finally, a total of 144 patients with metatarsalgia were included in the study. Of the total sample, 84 patients had RA (RAm), and 60 patients did not have RA (RAnm). There were 78 female patients in the RA group (92.8%) and 52 female patients in the nmRA group (86.6%) (*p* = 0.216). The mean age of the sample was 61.8 ± 12.1 in the mRA group and 61.4 ± 10.3 in the nmRA group (*p* = 0.811, ICC = −4.25/3.34). Body mass index was similar in both groups, with a mean of 25.6 ± 4.0 in the mRA group and 25.9 ± 4.8 in the nmRA group (*p* = 0.710, ICC = −1.18/1.74). Unilateral pain was present in 13 patients in the mRA group and 12 patients in the nmRA group. RA patients had a mean disease duration of 18 ± 14.44 years, and a median duration of 14 ± 14.36 years.

Data on medication, comorbidities, and habits are shown in Table 1.

### 3.1. Foot Pain and Functionality

The analysis of pain and FFI, both separated into the three subscales and as the total score, is shown in Table 2.

There were no statistically significant differences in the degree of perceived pain with either the VAS scale or the FFI pain subscale, but the RA group had greater disability and activity limitations. Pain was mostly from mixed components in the RA group and mechanical components in the non-RA group.

### 3.2. The Results of the Clinical Variables Provided the Following Data

#### 3.2.1. Joint Mobility

The evaluation of the mobility of the ankle, 1st MTPJ and STJ are shown in Table 3.

#### 3.2.2. Digital Deformities of Lesser Toes

LDD, MTPJ alterations, and their stiffness. Digital deformities, the presence of subluxation and luxation at the clinical level, and the flexibility of the latter, assessed through Kelikian’s test, are shown in Table 4.

#### 3.2.3. Digital Hallux Deformities

The deformities of the first ray are shown in Table 5. All the pathologies evaluated in the mRA group and all the degrees of HV were statistically significant.

#### 3.2.4. Foot Type by FPI

It was observed that the sample was very heterogeneous. The most frequent foot type was the neutral foot, and feet with alterations, both supinated and pronated, were more prevalent in mRA. The data are presented in Table 6.

#### 3.2.5. Radiology

A greater general presence of osteoarticular damage of the analysed variables was observed in mRA (erosions, JSR, global count of both subluxations and luxations), both from 1st to 5th MTPJ with IPJ of the hallux and from 2nd to 5th MPTJ, and a larger number of affected joints per foot. The radiological findings are shown in Table 7.

#### 3.2.6. Ultrasound

There were statistically significant differences in the amount of total synovitis found between mRA and nmRA at the ultrasound level. On the other hand, there was no difference in the evaluation of the 2nd to 5th joints, although there continued to be a larger number of MTPJ with synovitis in the mRA group. Five patients with mRA presented positive Doppler. The data are shown in Table 8.

#### 3.2.7. Total Count of Patients with Lesions and Number of Lesions

The global count of patients affected and the number of lesions are shown in Table 9.

## 4. Discussion

The patients with RA in remission continued to present with foot pain. This pain may be due to structural or biomechanical variables. Considering that the forefoot is one of the most affected areas in these patients [8,9,17,19] and the frequency of its affectation in the general population [38,39], this study was performed in patients with or without RA to identify the similarities and differences between these two groups. The literature shows studies that evaluated patients with or without RA with heterogeneous foot pain [19,23], although they do not indicate pain localisation [25] or have another pain localization [21], did not evaluate biomechanical or structural alterations [13], did not include a control group [6,16,21], or the control group was constituted by healthy participants [67]. 

Regarding pain, no differences were found in the degree of pain, neither through the VAS scale nor with pain FFI, although the patients with RA declared a greater consumption of analgesic and anti-inflammatory drugs, which could be due to the complex mechanisms related to pain in these patients, as well as to the inflammatory nature of the disease. In fact, the group with RA presented a statistically greater inflammatory component of pain (mixed and inflammatory pain). Our results are in line with those of González et al. [19], who also found greater mixed pain in a RA group with pain compared to a non-RA group with pain. 

In regard to disability, mobility limitation, and habits, several authors have observed that foot function was affected in individuals with RA [16,22] in general function, disability, and mobility limitation compared to the control group [22]. We also observed greater disability and mobility limitations in the mRA group, which is in line with these results. Moreover, metatarsal pain in the general population can also generate disability. Kuyvenhoven et al. [41] evaluated FFI in patients with non-traumatic pain in the forefoot, finding maximum values of disability of 19.8%. These results are still lower than those of our mRA group, corroborating the greater disability found in patients with RA. Furthermore, this group was more sedentary, which could be related to this greater limitation, as it especially affects walking [9] and, therefore, mobility limitation. 

A high prevalence of mobility limitations in the 1st MTPJ and DFTFTJ was observed in the two groups. These similarities could be due to the fact that both alterations in the first ray [68] and mobility limitations of the DFTFTJ [69,70] may be related to metatarsal pain. In addition, the limitations of the DFTFTJ could be associated with the mobility limitations of the MTP [71]. Other authors [6,19] in their sample with heterogeneous pain obtained greater mobility limitation in their RA group, which was lower than in our sample; this could also be explained by the specific localisation of pain in the forefoot in our study. Similarly, the mobility limitation of the subtalar joint (STJ) did not show differences between the two groups, presenting a lower frequency with respect to the previous limitations. No studies were found on the relationship of STJ limitation with metatarsalgia. 

No statistically significant differences were found with regard to lDD, indicating a high prevalence in both groups. LDDs are caused by metatarsal pain [72,73], which could also be related to the specific pain of the metatarsal area. Moreover, the limitations of the DFTFTJ may be associated with lDD. Other studies on non-specific foot pain in the metatarsal area [6,19] have found a lower amount of digital deformities (especially in the group without RA and with foot pain) than in our study, indicating that this is a characteristic related to pain localisation. Compared to other studies on RA [17] that do not indicate pain or studies with non-specific foot pain in the metatarsal area [6], we also found a greater proportion in our sample than in that of most of said studies. Reina Bueno [25] reported similar proportions, although no pain localisation was specified; thus, we cannot compare their results with ours.

In relation to the presence of subluxation/luxation and Kelikian’s test, the mRA group presented a larger number of subluxations and greater stiffness. However, our sample showed a lower prevalence than studies such as that of Siddle HJ et al. [20] with pain in the forefoot. The differences in the findings could be due to the fact that their diagnosis of RA is based on the criteria of 1987, whereas ours is based on those of 2010. Furthermore, subluxation is a characteristic that may generate pain in the general population, as well as stiffness. Several studies have demonstrated that hypomobility, contractures, and predislocation of the MTPJ produce pain, as well as an increase in plantar pressure in that localisation [74,75]. The fact that the RA group presented a greater proportion of these pathologies could be associated with factors of the disease, such as a previous acute inflammation [76], as well as with the medication used in patients with RA [77]. 

A high prevalence of first-ray pathology was observed, which was statistically significant in the RA group. The most prevalent pathology was HV, followed by HL and HR (although to a much lesser extent), which corroborates the findings of other studies on the high prevalence of HV in samples with RA [6,15,19,22]. Comparing our results with studies on the prevalence of HV in the general population, our percentages were greater, with 58.33% in our mRA group and 43.33% in our nmRA group, versus 23% [78] in young adults and 35.7% in people aged over 65 years, both data lower than those in our study. The same has been reported in studies with non-specific pain in the forefoot [19] or with previous pain in the hindfoot [21], with lower prevalence compared to our sample. This could be explained by the fact that the first-ray pathology is comprised of the causes of metatarsal pain [69,79]. On the other hand, differences were found between the patients with and without RA, which could be due to the fact that HV is also included among the consequences of RA, although having HV does not always generate a transfer of pressure to the adjacent rays [40,73,79], with other variables being more involved in the transfer of said pressure, such as the degree of HV [40]. In this case, the patients with RA have a greater degree of HV; thus, this difference could be the reason why HV is a frequent factor in the aetiology of metatarsal pain in this population, and this greater degree could be due to the characteristics of the disease. 

When valuing the FPI, we identified that most of the sample had neutral feet, although they were significantly more altered (supination, pronation) in the mRA group, with the supinated foot being the most frequent in both samples. Studies on FPI in patients with RA frequently report greater pronation [6,21,28], increasing with the evolution of the disease. One of the causes that could influence our results is, once again, the exclusive localisation of pain in the forefoot, as it would discard diagnoses such as posterior tibial tendon dysfunction (PTTD), which is widely related to greater pronation [80], as well as the specific removal of pain in the M1 head. Makoto Hirao [81] found that the patients with greater pronation had a lower degree of central metatarsal pain since the pressure was rather focused on the M1 head. In addition, the method selected to measure pronation could also be an influential factor [25].

The presence of synovitis was frequent in both groups, showing significant differences in all metatarsal heads and not only the pain localisation (2nd to 5th MTPJ). The fact that only the presence or absence of synovitis was evaluated could also imply differences that were not contemplated in this study. Van der Leeden [33] also found a high prevalence of inflammation and pain in patients with RA in metatarsophalangeal joints and pain in the forefoot. In our study, the patients without RA also presented inflammation and pain; thus, it could be a finding compatible with inflammation secondary to a mechanical cause. Furthermore, although there were only five patients with RA in remission with positive Doppler, this could also corroborate the importance of evaluating the foot joints that are not included in the DAS 28 [82]. Compared with other studies with heterogeneous pain [19], we observed a large amount of synovitis in general in the patients with metatarsal pain, with and without RA, greater than that of patients with heterogeneous pain with RA, thus it could be related to the specific metatarsal pain of the sample. Therefore, the presence of synovitis is a frequent characteristic in patients without RA, making it necessary to differentiate between synovitis caused by mechanical factors and other factors that are not related to the disease, which indicates the importance of identifying them and even considering them in the valuation of patients in remission. The fact that the patients with RA had greater synovitis in the 1st MTPJ could be due to the greater presence of first-ray pathologies in this population. 

In simple radiology, we observed a larger amount of erosion in the RA group, with this sign being a criterion used even for the diagnosis of the disease [83]. Although JSR is gathered within the joint pain grading scales in RA, this condition could occur in the general population due to mechanical causes [11]. In this case, it was also significant in the RA group. This could be related to the statistically significant metatarsophalangeal alterations found in this group. The fact that there was a lower amount of osteoarticular damage in the RA patients, with both groups presenting a similar degree of pain, could indicate the importance of the mechanical variables and morphostructural alterations in the generation of metatarsal pain in the patients, with and without RA, and the importance of their evaluation. In fact, studies similar to that of Borman et al. [10] did not detect a relationship between the radiological scores and foot pain at the time of valuation or with the duration of the symptoms. In addition, the fact that the patients with RA had greater osteoarticular damage could be associated with the disease, as indicated in studies that found greater osteoarticular damage along the evolution of RA [36].

With regard to the number of lesions found, studies such as that of Adja Bal 2006 [22] with a group of patients with RA and another group without RA, where the presence of pain was not indicated, reported that the difference between the groups was that the RA group presented more than one joint and tendon affected, which corroborates the systemic nature of RA. In our study, we found similar conclusions in patients with metatarsal pain, since patients with metatarsal pain without RA showed a high percentage of foot affectation. However, in the analysis of the number of damaged joints or present pathologies, we identified a greater number in the patients with RA. On the other hand, although the degree of pain was similar, we did not find the same type of pain, the same disability, mobility limitation, or the same need for analgesic medication, which could also be associated with the variables that differentiate them and with the disease itself.

The fact that metatarsalgia presents similarities in both groups could be due to the existence of biomechanical factors of the foot, which may be influencing the patients with RA in remission; thus, it would be necessary to conduct a biomechanical exploration of the foot of these patients.

This study opens new research lines that may specifically evaluate the influence of the structural and biomechanical alterations of the foot in this type of patient, as well as the importance of considering the characteristics of the foot when evaluating the localisation of pain and/or inflammation.

## 5. Conclusions

1. Although the patients with RA in remission with metatarsalgia had a greater component of inflammatory pain, the degree of pain recorded with the VAS and FFI pain scales did not differ with respect to the nmRA group. Therefore, it seems that the degree of pain does not depend on presenting RA in remission, taking into account that the perception of pain has a subjective component and that complex mechanisms could be involved.
2. We observed greater disability and FFI limitation in the mRA group in remission with metatarsal pain with respect to the nmRA group, in addition to an increase in sedentary lifestyle, which could be related to greater osteoarticular damage, valued with the Sens scale (JSR and erosions), as well as an increase in the number of subluxations and luxations, greater stiffness in lDD and their MTPJ, and a larger number of affected joints in each foot, even with a similar degree of pain.
3. Similarities were detected between the mobility of the 1st MTPJ and DFTFTJ since they were widely limited in both groups. The finding of these similarities could be associated with the relationship between these two characteristics and the common metatarsal pain in both groups.
4. No differences were found in the presence of lDD; thus, it seems that they do not depend on the activity of RA but on the aftereffects and possible mechanical causes that affect both groups and generate metatarsalgia.
5. Synovitis to an unspecified degree does not depend on presenting RA in remission, although a positive Doppler signal could be present in patients in remission; thus, it would be necessary to evaluate the MTPJ that is not included in the DAS 28.

## 6. Limitations

The footwear of the patients was not taken into account, and it could be involved in both the presence of pain and in the development of some deformities, such as HV and digital deformities.

Only the presence or absence of deformities and osteoarticular damage was considered, disregarding their degree, which could show more differences in the results.

We did not contemplate the fact that patients with mRA could present the variable “fear of moving”, which could influence the limitations of activities of daily living and the sedentary lifestyle found in this study.

Similarly, the professional activity of the patients was not considered.

There are more variables that could be related to metatarsal pain that were not included in this study, and that would be interesting to evaluate in future studies, as well as their relationship with foot pain and function.

Although we sought to ensure that there were no statistically significant differences in age and gender, ideally the sample should be evenly matched.

## 7. Strengths and Weaknesses

This is a novel study as it includes patients with the same pain location, which may provide evidence for the biomechanical exploration of the patients and the use of both conservative and surgical therapies to eliminate their pain symptomatology.

There were several strengths of this study: There was a large sample of patients, with no statistically significant differences in age or sex, and with the same pain location. With regard to the evaluation of biomechanical, ultrasound, and radiological variables. The researchers evaluating the ultrasound scans and radiographs were blinded. All the researchers had extensive clinical experience, and the scans were always performed by the same researcher.

Unfortunately, there were also several weaknesses to this study. The sample was not fully matched and consisted fully of patients from communities in Madrid (Spain). In addition, there were more variables that we did not consider that should be studied further in future studies, and the researcher performing the clinical tests was not blinded.

## Figures and Tables

**Table 1:** Age, BMI (body mass index), medication, comorbidities, and habits.

	mRA * (*n* = 84)		nmRA ** (*n* = 60)		
Medication	*n* Patients	%	*n* Patients	%	*p*
NSAIDS ***	39	46.4	13	21.6	0.002
Paracetamol	12	14.2	5	8.3	0.275
Steroid	40	47.6	0	0	0.000
Fames	63	75	0	0	0.000
Methrotexate	41	48.8	0	0	0.000
Biological	33	39.2	0	0	0.000
Vit D	24	28.5	3	5	0.000
Calcium	20	23.8	3	5	0.002
Antiaggregants	4	4.7	6	10	0.223
AHT ****	23	27.3	18	30	0.731
Cholesterol	31	36.9	29	48.3	0.170
Hypothyroidism	17	20.2	9	15	0.420
Habits	*n* patients	%	*n* patients	%	*p*
Smoking	18	21.4	18	30	0.471
Alcohol	2	2.3	3	5	0.333
Sedentarism	41	48.8	11	18.3	0.001

mRA *: group with metatarsal pain and rheumatoid arthritis diagnosis in remission. nmRA **: group without rheumatoid arthritis and with metatarsal pain. NSAIDS ***: non-steroidal anti-inflammatory drugs. AHT ****: arterial hypertension. n: number of patients.

**Table 2:** VAS pain scale and FFI (Foot Function Index).

Pain and FFI	mRA (*n* = 84)		nmRA (*n* = 60)			
	Mean	sd	Mean	sd	*p*	ICC95%
VAS pain [43]	7.3	2.1	6.7	2.1	0.097	(−1.27/0.10)
FFI pain * [46]	75.9	20.3	70.5	20.0	0.131	(−1.24/−0.16)
FFI disability **	32.6	26.4	11.1	14.1	0.000	(−2.89/−1.41)
FFI activity Limitation ***	23.8	24.5	04.6	05.3	0.000	(−2.52/−1.31)
FFI total ****	59.7	29.1	32.7	15.9	0.000	(−3.54/−1.86)
Pain time	*n* patients	%	*n* patients	%	*p*	
Acute	3	3.5	3	5	0.672	
Type of pain	*n* patients	%	*n* patients	%	*p*	
Mechanical	29	34.5	45	75	0.000	
Inflammatory	7	8.3	0	0		
Mixed	43	51.1	15	25		
Other	5	5.9	0	0		

FFI pain *: (subscale of pain in the FFI questionnaire). FFI disability **: (subscale of disability of the FFI questionnaire). FFI activity limitation ***: (subscale of activity limitation of the FFI questionnaire). FFI total ****: (FFI questionnaire).

**Table 3:** Joint mobility.

Joint Mobility	mRA (*n* = 84)		nmRA (*n* = 60)		
	*n* patients	%	*n* patients	%	*p*
Ankle *, DFTFTJ					
Normal	22	26.2	14	23.3	0.696
Limited	63	75%	46	76.6	
1st MTPJ mobility **					
Normal	28	33.3	20	33.3	0.791
Limited	49	48.3	33	55	
Stiff	7	8.3	7	11.6	
FHL test ***					
Positive	64	76.2	45	75	0.870
STJ ****					
Limited	30	35.7	12	20	0.119

Articular mobility. Ankle *, DFTFTJ (dorsiflexion of the tibiofibular-talar joint), dorsal flexion mobility of the ankle with a flexed and/or extended knee evaluated with Silfverskiöld’s test. DF1st MTPJ **: (first metatarsophalangeal joint mobility). FHL test ***: (functional hallux limitus test). STJ (subtalar joint) ****: mobility.

**Table 4:** Lesser-toe deformities. Subluxation and luxation of metatarsophalangeal joints. Kelikian’s test.

Lesser Toes	mRA (*n* = 84)		nmRA (*n* = 60)		
	*n* patients	%	*n* patients	%	*p*
DD *	59	70.2	34	56.6	0.093
Subluxation **	49	58.3	17	28.3	0.000
Kelikian’s test	*n* patients	%	*n* patients	%	*p*
Flexible	28	33.3	35	58.3	0.009
Semiflexible	35	41.6	18	30	
Stiff	21	25	7	11.6	

DD *: (digital deformities of lesser toes). Subluxation **: (subluxation and luxation of lesser toes).

**Table 5:** Digital deformities. Hallux.

Hallux	mRA (*n* = 84)		nRAm (*n* = 60)		
	*n* patients	%	*n* patients	%	*p*
Deformity	69	80.9	34	56.6	0.093
Type of deformity	*n* patients	%	*n* patients	%	*p*
HV *	49	58.3	26	43.3	0.045
FHL/SHL **	12	17.8	7	15	
HR ***	6	5.9	3	5	
Others	2	2.4	0	0	
Manchester HV ****					
0	34	40.4	34	56.6	
I	26	30.9	19	31.6	0.048
II/III	23	21.3	7	11.6	

HV *: (hallux valgus). FHL **: (functional hallux limitus). SHL **: (structural hallux limitus). HR ***: (Hallux rigidus). Manchester HV ****: (Manchester scale of hallux valgus).

**Table 6:** Type of foot. FPI (Foot Posture Index).

Type of Foot	mRA (*n* = 84)		nmRA (*n* = 60)			
FPI	*n* patients	%	*n* patients	%	*p*	
Neutral	40	47.6	38	63.3	0.165	
Pronated	16	19.1	7	11.6		
Supinated	28	33.3	15	25		

**Table 7:** Radiologý.

Radiology	mRA (*n* = 84)		nmRA (*n* = 60)			
JSR affectation	Mean	sd	Mean	sd	*p*	ICC 95%
1st to 5th MTPJ * + IPJ hallux **	3.63	2.1	1.5	1.4	0.000	(−2.59/−1.42)
2nd to 5th MTPJ *	2.439	1.7	0.7	1.2	0.000	(−2.07/−1.06)
nº MTPJ * affected 2nd to 5th JSR ***	*n* patients	%	*n* patients	%		
0	20	23.8%	36	60%		
1	12	14.3%	9	15%		
2	4	4.7%	8	13.3%		
3	4	4.7%	1	1.6%		
4	42	50%	4	6.6%		
Affectation erosions	mRA		nmRA			
	Mean	sd	Mean	sd	*p*	
1st to 5th MTPJ * + IPJ hallux **	2.5	1.7	0.1	0.3	0.000	(−2.87/−2.10)
2nd to 5th MTPJ	1.9	1.4	0.1	0.3	0.000	(−2.19/−1.56)
nº erosions 2nd to 5th MTPJ *	*n* patients	%	*n* patients	%		
0	13	15.5%	52	86%		
1	24	28.6%	5	8.33%		
2	17	20.2%	0	0		
3	11	13.1%	0	0		
4	18	21.4%	0	0		
SENS scale total. JSR *** + erosions	6.1	3.2	1.6	1.3	0.000	(−2.87/−2.10)
Luxations or subluxations 2nd to 5th MTPJ *	Mean	sd	Mean	sd	*p*	
Subluxations/luxations 2nd to 5th	2.0	1.6	0.7	1.1	0.000	(−1.76/−0.84)
nº MTPJ * affected 2nd to 5th subluxations/luxations	*n* patients	%	*n* patients	%		
0	23	27.4	36	60		
1	14	16.6	13	21.6		
2	10	11.9	6	10		
3	8	9.5	2	3.3		
4	27	32.1	3	5		

MTPJ *: metatarsophalangeal joint. IPJ hallux **: interphalangeal joint of the hallux. JSR ***: joint space reduction.

**Table 8:** Ultrasound.

Ultrasound	mRA (*n* = 84)		nmRA (*n* = 60)			CI 95%
	Mean	sd	Mean	sd	*p*	
MTPJ * 1st to 5th	2.50	1.6	1.9	1.3	0.041	(−1.01/0.02)
MTPJ * 2nd to 5th	2.02	1.4	1.5	1.2	0.054	(−0.88/0.006)
nº MTPJ * affected 2nd to 5th	*n* patients	%	*n* patients	%	*p*	
0	17	20.2	15	25		
1	15	17.8	13	21.6		
2	17	20.2	18	30		
3	19	22.6	10	16.6		
4	16	19.1	4	6.6		
Some synovitis MTPJ * 2nd to 5th	mRA (*n* = 84)		nmRA (*n* = 60)			
	*n* patients	%	*n*	%	*p*	
Without doppler	61	72.6	42	70	0.120	
With doppler	5	5.9	0	0	0.001	

MTPJ * (metatarsophalangeal joint).

**Table 9:** Patients affected by synovitis in ultrasound and osteoarticular damage in radiology. Total number of ultrasound and radiological lesions.

Patients with Synovitis (Ultrasound) and Osteoarticular Damage (Radiology)	mRA (*n* = 84)		nmRA (*n* = 60)	
Synovitis, subluxations, erosions, JSR	*n* patients	%	*n* patients	%
Synovitis (ultrasound)	67	79.7	45	75
Subluxation/luxation	49	58.3	17	28.3
Erosions	71	84	4	6.6
JSR *	62	73.8	24	40
Total nº of lesions	MTPJ ** affected/total	%	MTPJ ** affected/total	%
Synovitis	170/336	50.6	95/240	39.6
Subluxations/luxations	166/336	49.4	49/240	20.4
Erosions	163/336	48.5	5/240	2.1
JSR	200/336	59.5	44/240	18.3

JSR *: joint space reduction. MTPJ **: metatarsophalangeal joint.

## Data Availability

The datasets used and/or analyzed during the current study are avail-able from the corresponding author upon reasonable request.
